# Effect of *Perilla frutescens* Fixed Oil on Experimental Esophagitis in Albino Wistar Rats

**DOI:** 10.1155/2013/981372

**Published:** 2013-08-20

**Authors:** Ekta Arya, Sudipta Saha, Shubhini A. Saraf, Gaurav Kaithwas

**Affiliations:** Department of Pharmaceutical Sciences, Babasaheb Bhimrao Ambedkar University (Central University), Vidya Vihar, Rai Bareli Road, Lucknow 226025, India

## Abstract

The present study was undertaken to elucidate the effect of *Perilla frutescens* fixed oil on experimental esophagitis in albino rats. A group of rats (*n* = 6), treated with control vehicle (0.9% NaCl in double distilled water, 3 mL/kg, i.p.) and *Perilla frutescens* fixed oil (100%) (1, 2, and 3 mL/kg, i.p.), or pantoprazole (30 mg/kg, i.p.), were subjected to pylorus and forestomach ligation. Animals were sacrificed after 6 h and evaluated for the gastric pH, volume of gastric juices, total acidity, esophagitis index and free acidity. Esophageal tissues were further subjected to estimations of TBARS, GSH, catalase, and SOD. Treatment with fixed oil significantly inhibited the gastric secretion, total acidity, and esophagitis index. The oil also helped to restore the altered levels of oxidative stress parameters to normal. The present study also makes evident the *in vitro* antihistaminic and anticholinergic activity of alpha linolenic acid (ALA) (18 : 3, *n* − 3) on isolated rat ileum preparation. The lipoxygenase inhibitory, histamine antagonistic, antisecretory (anticholinergic), and antioxidant activity of the oil was attributed for its efficacy in reflux esophagitis.

## 1. Introduction

Gastroesophageal reflux disease (GERD) is delineated as a condition that springs up due to reflux of gastric contents into the esophagus leading to mucosal damage and oxidative stress; the condition may be asymptomatic or result in symptoms. The commonness of GERD is estimated to be 10–20% in western countries, making it one of the most predominant gastrointestinal disorders [1]. Recent investigations have reported that the mucosal damage in GERD is due to handful inductive agents in the refluxate [2] that galvanizes the mucosal and submucosal cells to release mediators, eliciting anti-inflammatory reaction, leading to visceral hypersensitivity and other symptoms of GERD [3]. Inflammatory process insinuates to play a key role in the inherent mechanisms of the symptoms and pathogenesis of other gastrointestinal conditions including functional dyspepsia and irritable bowel syndrome [4].

The plant *Perilla frutescens* Linn belongs to the family Labiatae and is also called as shiso plant or beefsteak plant purple mint [5]. The seeds produce a light yellow colored fixed oil with strong taste whose intensity might be compared to that of mint or fennel. The oil contains unsaturated fatty acids like oleic acid (18 : 1; n-9) (14–23%), linoleic acid (LA) (18 : 2; n-6) (11–16%), and alpha-linolenic acid (ALA) (18 : 3, n-3) (54–64%) [6]. The oil has been divulged to have antiasthmatic, antibacterial, antipyretic, antiseptic, antispasmodic, antitussive, emollient, expectorant, and antioxidant activity [7]. In preceding studies, the fixed oil obtained from *Linum usitatissimum *fixed oil has illustrated antiulcer activity in variable models [8] and efficacy in experimental reflux esophagitis (RE) due to the presence of high amounts of ALA (18 : 3, n-3) [9]. In consideration of the antecedent reports, the present work was commenced to evaluate the possible effects of *Perilla frutescens* fixed oil (PFO) on experimentally induced esophageal lesions in animal models. To delve into the possible role of ALA (18 : 3, n-3), the anticholinergic and antihistaminic activity of ALA (18 : 3, n-3) was investigated *in vitro*, using isolated rat ileum.

## 2. Experiment

### 2.1. Materials

The seeds were procured from the local market of Kanpur and authenticated at National Botanical Research Institute, Lucknow, India. A voucher specimen of the same was submitted for future reference (NBRI/CIF/299/2012). Pantoprazole was procured from Alembic Chemical Pvt Ltd (India). ALA (18 : 3, n-3) (CAS-506-26-3) was procured from Sigma Aldrich, USA. All other chemicals were purchased from S.D. Fine Chemicals, Mumbai, India. 

### 2.2. Extraction of Oil

**Figure 1:**
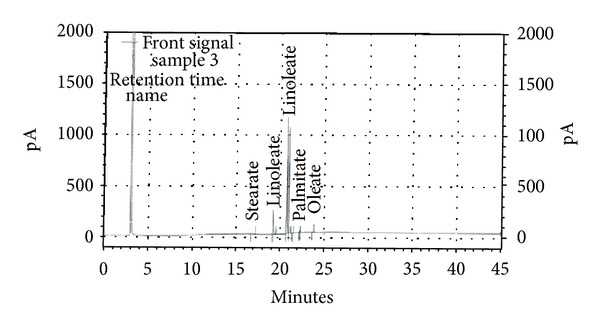
Gas liquid chromatographic analysis of *Perilla frutescens *fixed oil.

Seeds were crushed and cold macerated in petroleum ether (40–60°C) for 7 days. The seeds were washed, dried, crushed, and weighed, before extraction. Petroleum ether was evaporated from the extract and oil was filtered to clarity. The yield of fixed oil was 25.50% v/w with reference to dried seeds. The oil thus obtained was subjected to gas chromatographic analysis by methyl esterification. The gas chromatographic analysis (Agilent GC make 6890; Column B.P. 225 50 m capillary column), using Detector FID 250C, carrier gas: nitrogen, injection volume 1 *μ*L, and internal standard: cetyl alcohol of the methyl ester of oil, affirmed the presence of LA (18 : 2; n-6) (16.94%) and ALA (18 : 3, n-3) (68.21%) as major constituents (Figure 1). The oil was stored at room temperature in amber-colored airtight bottle. To avoid oxidation, the oil was filled to the brim of the bottle so that there was no head space.

### 2.3. Animals

Wistar strain albino rats (175–200 g) were obtained from Indian Veterinary Research Institute, Animal Resources, Bareilly (UP). Animals were housed under standard conditions of temperature (25 ± 1°C) with 12 h light/dark cycle and had a free access to commercial pellet diet and water *ad libitum*. The animals were given a week to get acclimatized with the laboratory condition, before experimentation. The study was commended by the Institutional Animal Ethics Committee (Reg. no. 1279/AC/09/CPCSEA.).

### 2.4. Induction of Reflux Esophagitis

**Figure 2:**
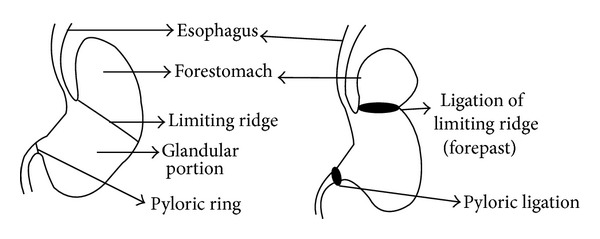
Procedure for ligation of stomach.

Groups of rats (*n* = 6), fasted for 24 h received normal saline (3 mL/kg, i.p.) (Sham control), vehicle (normal saline, 3 mL/kg, i.p.), PFO (100%) (1, 2, 3 ml/kg, i.p.), or pantoprazole (30 mg/kg, i.p.) (operated groups). After 1 h, celiotomy was performed under pentobarbitone sodium anesthesia (50 mg/kg, i.p.) and RE was induced (except in sham control) by ligating the forestomach and corpus with 2-0 silk suture and pylorus ligation as per the method described by Renu et al. [9] (Figure 2). After 6 h, the animals were sacrificed by cervical dislocation and the chest was opened with a midline incision. The tissue esophagus and stomach were removed. The stomach was opened along the greater curvature and the esophagus was dissected out by extending the dissection line along the major axis to determine the esophagitis index [9]. The severity of esophagitis was calculated in Table 1.

The sum of scores was divided by a factor of ten which was designated as the esophagitis index. The volume of gastric juices was measured as described subsequently under “gastric secretion in pylorus ligated rats” [8]. The pH measurement of gastric juice was done using a pH meter.

### 2.5. Estimation of Free Radical Generation

Esophageal tissue was minced well, homogenized in ice-cold 0.01 M Tris-HCl buffer (pH 7.4), and subjected to the estimations of thiobarbituric acid reactive substances (TBARS) [10], tissue glutathione (GSH) [11], catalase [12], and superoxide dismutase (SOD) [13]. 

### 2.6. *In Vitro* Anticholinergic and Antihistaminic Activity

A cumulative concentration response curve (CRC) for acetylcholine (Ach) and histamine (1–256 *μ*M) was recorded on a 4-minute time cycle (a 30-second base line, followed by 30 sec of contact time and three subsequent washings at 1-minute interval) using isolated rat ileum preparation. The Ach and histamine were added to the bath in geometric progression (10 *μ*g/mL stock solution; bath concentration from 1 to 256 *μ*M), until a maximal effect was obtained. In addition, three further sets of tissues were washed for 30 min with Tyrode solution and allowed to equilibrate for 15 min in the presence of three different concentrations of ALA (18 : 3, n-3) (0.1, 0.2, and 0.3 mL). Cumulative CRCs for Ach and histamine (1–256 *μ*M) were then plotted for each of the three concentrations of ALA (18 : 3, n-3). All of the experiments were performed in triplicate (a total of twelve ilea from separate animals for each set) [14].

The responses to Ach and histamine were converted to a percentage of the maximal contractile response. EC_50_ values for Ach and histamine were calculated in the absence or presence of the different concentrations of ALA (18 : 3, n-3). Concentration ratios for the antagonist were determined by dividing the EC_50_ values for the agonist, in the presence of the antagonist, by the EC_50_ values in the absence of the antagonist. An Arunlakshana-Schild plot, showing log (concentration ratio-1) versus log molar concentration of antagonist, was constructed by using the data. The pA_2_ value was determined by linear regression analysis [15, 16].

### 2.7. Statistical Analysis

All data were presented as mean ± SD and analyzed by one way ANOVA followed by Bonferroni test for the possible significance identification between the various groups. Statistical significance was considered to control (^a^ *P* < 0.05, ^b^ *P* < 0.01, ^c^ *P* < 0.001) and toxic control (^x^ *P* < 0.05, ^y^ *P* < 0.01, ^z^ *P* < 0.001). Statistical analysis was carried out using Graph pad prism 3.0, San Diego, USA.

## 3. Results 

The present investigation was endeavored to determine any antiesophagitis effects of PFO in a rat model of RE induced by pylorus and forestomach ligation. The toxic control group was accorded with induced esophageal inflammation, edema, and ulcer. Intraperitoneal administration of PFO (1, 2, and 3 mL/kg) significantly averted the RE in a dose-dependent manner. PFO (3 mL/kg) significantly inhibited the esophagitis index (35.37%), gastric volume (67.64%), and total acidity (60.12%) in comparison with vehicle control (Table 2). Pantoprazole produced 46.34% inhibition of esophagitis index consequently. As depicted in Table 3, levels of tissue SOD and catalase were significantly decreased in the toxic control animals (i.e., vehicle control), whereas TBARS and GSH levels were observed to be increased in sham control group. The intraperitoneal administration of PFO significantly altered the levels of SOD, catalase, GSH, and TBARS in a dose-dependent manner. 

**Figure 3:**
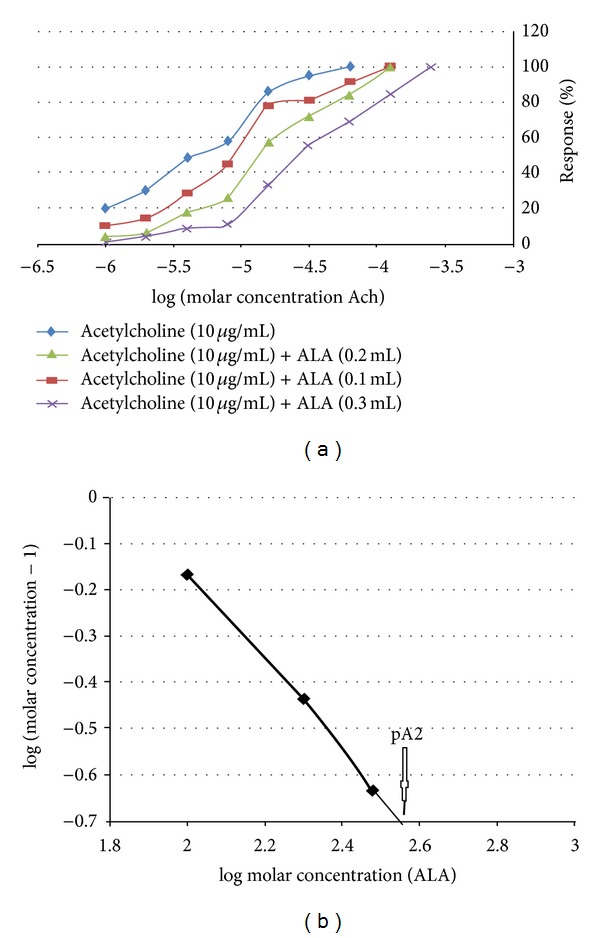
Effects of acetylcholine (1–256 *μ*M) on isolated rat ileum preparation in the presence and absence of 0.1, 0.2, and 0.3 mL of alpha-linolenic acid (ALA). In (a) data are expressed as the mean + SEM; *n* = 3. Ach = acetylcholine. (b) shows the Arunlakshana-Schild plot of data from graph (a).

**Figure 4:**
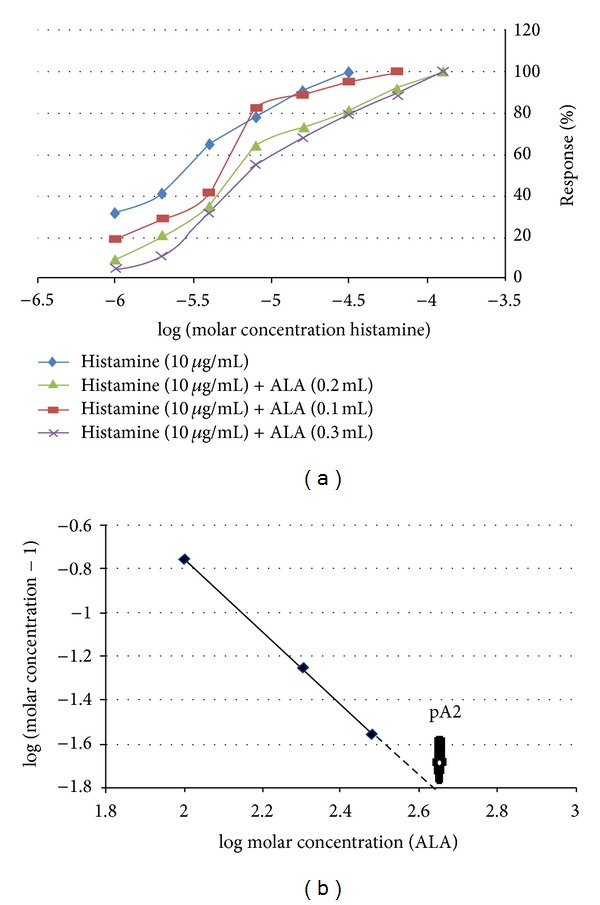
The effects of histamine (1–256 *μ*M) on isolated rat ileum preparation in the presence and absence of 0.1, 0.2, and 0.3 mL of alpha-linolenic acid. In (a), data are expressed as the mean + SEM; *n* = 3. Ach = acetylcholine. (b) shows the Arunlakshana-Schild plot of data from graph (a).

Ach and histamine elicited a concentration dependent increase in the contractile response of isolated ileum preparations. ALA (18 : 3, n-3), which was brought to bear as an antagonist in this study, produced a parallel right shift of the both Ach and histamine concentration-response curves without any detraction in the utmost response. The parallel right shift illustrates the competitive nature of the antagonism (Figures 3(a) and 4(a)). Analysis of the Arunlakshana-Schild plot bestowed the slope, close to unity, which over and over confirmed the competitive nature of ALA (18 : 3, n-3) antagonism on cholinergic and histaminergic receptors in the present experiment (Figures 3(b) and 4(b)). The pA_2_ values of ALA (18 : 3, n-3) was found to be 2.56 for acetylcholine and 2.65 for histamine by using the Arunlakshana-Schild plot. The pA_2_ value is an indicator of antagonist specificity; high values depict high specificity, whereas lower values imply a lower specificity of the antagonist. The pA_2_ value of 2.56 and 2.65, which we accomplished in our experiments, are considered significant. The results therefore suggest that ALA (18 : 3, n-3) exhibits significant antagonist specificity for the cholinergic and histaminergic receptors.

## 4. Discussion

The present study conceded that the PFO exhibits significant protection against the RE in experimental animals following intraperitoneal administration. Ligation of the forestomach and pyloric end propagated the erosive and/or ulcerative type of lesions. The damage produced during the process could be fundamentally accredited to the reflux of gastric content containing significant amount of pepsin (proteolytic enzyme). Presence of acid also exaggerates the ulcer formation by its corrosive action thus keeping a peerless environment for pepsin activity [17, 18]. Nonetheless, intraperitoneal administration of PFO, in the present experiment, significantly decreased the gross volume of gastric juice secretion, total acidity, and esophagitis index and raised the gastric pH in comparison to control.

The antiulcer activity of the *Linum usitatissimum* fixed oil (LUFO) is available against NSAIDs, alcohol, histamine, reserpine, serotonin, and stress induced ulcers. The fixed oil also demonstrated antisecretory effect. The antiulcer activity of the LUFO was attributed to the lipoxygenase inhibitory, histamine antagonistic, and antisecretory (anticholinergic) activity and the same was accredited to the presence of ALA (57.38%, 18 : 3, n-3) [8]. The LUFO also demonstrated significant antihistaminic and anticholinergic activity against *in vitro* test [8]. Since PFO used in the present experiment contains higher concentrations of ALA (68.21%, 18 : 3, n-3), one would expect similar biological activity from PFO. However, to confirm the hypothesis that ALA (18 : 3, n-3) contributes towards the beneficial effects of PFO in the present study, efforts were made to evaluate the anticholinergic and antihistaminic activity of ALA (18 : 3, n-3). The study makes evident the *in vitro* antihistaminic and anticholinergic activity of ALA (18 : 3, n-3) on isolated rat ileum preparation.

The fixed oil obtained from *Perilla frutescens* contains ALA (68.21%, 18 : 3, n-3) and previous reports in the literature have divulged ALA (18 : 3, n-3) as a dual inhibitor of arachidonic acid metabolism; that is, it inhibits both the cyclooxygenase and lipoxygenase pathway [19]. Henceforth, decrease in gross volume of gastric juice secretion, total acidity, and esophagitis index by PFO could be congregately accredited to the lipoxygenase inhibitory, histamine antagonistic, and antisecretory (anticholinergic) effects of ALA (18 : 3, n-3).

Marked increase in gastric TBARS activity was ascertained in the toxic control in comparison to intact control. Our results suggest that RE produces free radical species that attack lipid components leading to lipid peroxidation and consumption of GSH in the first few hours of oxidative stress, directing diminished GSH level. Concomitant administration of the PFO significantly inhibited the free radical induced damage, to restore the altered TBARS and GSH levels. The restoration of TBARS level was dose independent. The MDA generation after the standard pantoprazole treatment was almost similar to toxic control which suggests the possible variability in the TBAR's generation after treatment. 

The enzymatic antioxidant activities of the CAT and SOD in esophageal tissue have been significantly decreased in the toxic control animals as compared with the sham control. Intraperitoneal administration of PFO significantly reverted these enzymes to near normal values. SOD by scavenging the superoxide radical generates hydrogen peroxide and molecular oxygen. Catalase existing in the cells catalyses the dismutation of hydrogen peroxide (produced due to scavenging effect of SOD) to water and molecular oxygen. The diminished levels of SOD and catalase in the present experiment could be accredited to be increased oxidative stress, accompanying increased consumption. Previous studies on PUFAs (polyunsaturated fatty acids, LA (18 : 2; n-6), and ALA (18 : 3, n-3)) have evidenced protection against oxidative stress by increasing the levels of several cellular antioxidants such as ascorbic acid, alpha-tocopherol, and GSH [20]. Baydas et al. also implied that the diet supplemented with antioxidants like ALA (18 : 3, n-3) exhibits significant antioxidant activity in rats [21]. Thus antioxidant activity ascertained in the present study could be attributed to the presence of ALA (18 : 3, n-3) (68.21%) and LA (16.94%) in PFO.

We would also like to mention that recently published studies have enumerated the effect of short supplementation of omega-3 fatty acids on postoperative ileus and GI motility in experimental animals. Wehner et al. (2012) [22] have enumerated the increase in GI motility after short-term omega-3 fatty acid supplementation and the same was attributed to the reduced neutrophil and nitric oxide levels, along with improved jejunal contractility and GI time. Another study published by Zhang et al. (2011) [23] demonstrates that the omega-3 fatty acids can stimulate the GI motility, attributed to the decreased levels of proinflammatory cytokinin production. It would be worth to mention that anti-inflammatory, antiarthritic, and antioxidant potential of the *L. usitatissimum* fixed oil (containing linolenic acid as a major constituent) has been already reported [24, 25]. 

From the lines of evidence, one would like to accomplish that the protective effect of PFO against RE could be attributed to the antisecretory (anticholinergic, antihistaminic), antioxidant, and lipoxygenase inhibitory activities due to the presence of ALA (18 : 3, n-3). To the best of our acquaintance, the present experimental data is the first to establish the anticholinergic and antihistaminic activity of ALA (18 : 3, n-3) along with efficacy of PFO against RE. It would be worthy to mention that the studies have reported significant anti-inflammatory property of PFO. Hence, the drug having activity against RE could be of great therapeutic concern as most of the anti-inflammatory drugs used in modern day medicine are ulcerogenic. Nevertheless, studies are required to inculcate the usability of PFO in RE.

## Figures and Tables

**Table 1:** 

Erosion (mm)	Score
1 or less	1
1-2	2
>2	3

**Table 2:** Effect of *Perilla frutescens* fixed oil (PFO) on pH, volume of gastric juice, total acidity, esophagitis index, and free acidity on experimental esophagitis in albino rats.

Groups	Treatment	pH	Volume of gastric juices (mL/100 g)	Total acidity (mEq/L)	Esophagitis index	Free acidity (mEq/L)
Group-I	Sham control (normal saline, 3.0 mL/kg, i.p.)	3.52 ± 0.02	3.65 ± 0.63	68.4 ± 0.95	0.49 ± 0.06	59.9 ± 0.42
Group-II	Toxic control (normal saline, 3 mL/kg, i.p.)	2.66 ± 0.01	6.86 ± 0.65	176.3 ± 0.85	0.82 ± 0.01	165.6 ± 0.52
Group-III	PFO (1 mL/kg, i.p.)	2.79 ± 0.11^c,x^	4.95 ± 0.77^b,z^ (27.84)	112.7 ± 0.11^c,z^ (36.07)	0.76 ± 0.007^c,y^ (7.31)	121.0 ± 0.70^c,z^ (26.93)
Group-IV	PFO (2 mL/kg, i.p.)	3.32 ± 0.08^c,z^	3.06 ± 0.33^z^ (55.39)	92.4 ± 0.68^c,z^ (47.58)	0.64 ± 0.008^c,z^ (21.95)	89.0 ± 0.54^c,z^ (46.27)
Group-V	PFO (3 mL/kg, i.p.)	3.75 ± 0.03^c,z^	2.22 ± 0.38^b,z^ (67.64)	70.3 ± 0.86^b,z^ (60.12)	0.53 ± 0.006^a,z^ (35.37)	56.0 ± 0.33^c,z^ (66.18)
Group-VI	Pantoprazole (30 mg/kg, i.p.)	3.91 ± 0.07^c,z^	1.36 ± 0.52^c,z^ (80.17)	73.7 ± 0.52^c,z^ (58.19)	0.44 ± 0.005^a,z^ (46.34)	61.30 ± 0.33^c,z^ (62.98)

Each group contains 6 animals. All data were presented as mean ± SD, and values in parenthesis represent percentage inhibition.

Statistical significance compared to control [^a^ *P* < 0.05, ^b^ *P* < 0.01, ^c^ *P* < 0.001] and toxic control [^x^ *P* < 0.05, ^y^ *P* < 0.01, ^z^ *P* < 0.001] using one way ANOVA followed by Bonferroni test.

**Table 3:** Effect of *Perilla frutescens* fixed oil (PFO) on antioxidant enzymes in esophageal tissues.

Groups	Treatment	Superoxide dismutase (unit of SOD/mg of protein)	Thiobarbituric acid reactive substances (nm of MDA/mg of protein)	Catalase (nm at H_2_O_2_/min/mg of protein)	Glutathione (GSH) (mg %)
Group-I	Sham control (normal saline, 3.0 mL/kg, i.p.)	1.00 ± 0.2	1.30 ± 0.18	18.85 ± 2.32	1.68 ± 0.05
Group-II	Toxic control (normal saline, 3 mL/kg, i.p.)	0.75 ± 0.11	2.94 ± 0.08	7.37 ± 0.40	0.94 ± 0.15
Group-III	PFO (1 mL/kg, i.p.)	0.83 ± 0.20	0.90 ± 0.09^c^	10.81 ± 0.75^c,x^	1.33 ± 0.13^c,z^
Group-IV	PFO (2 mL/kg, i.p.)	0.91 ± 0.05	1.36 ± 0.03^c,z^	15.57 ± 0.90^a,z^	1.46 ± 0.08^b,z^
Group-V	PFO (3 mL/kg, i.p.)	1.00 ± 0.15	1.37 ± 0.03^c,z^	19.60 ± 3.25^z^	1.50 ± 0.15^z^
Group-VI	Pantoprazole (30 mg/kg, i.p.)	1.25 ± 0.21^z^	2.70 ± 0.07^b,z^	23.81 ± 0.65^c,z^	1.61 ± 0.06^z^

Each group contains 6 animals. All data were presented as mean ± SD. Statistical significance compared to control [^a^ *P* < 0.05, ^b^ *P* < 0.01, ^c^ *P* < 0.001] and toxic control [^x^ *P* < 0.05, ^y^ *P* < 0.01, ^z^ *P* < 0.001] using one way ANOVA followed by Bonferroni test.
